# Longitudinal active living research to address physical inactivity and sedentary behaviour in children in transition from preadolescence to adolescence

**DOI:** 10.1186/s12889-015-1822-2

**Published:** 2015-05-17

**Authors:** Nazeem Muhajarine, Tarun R Katapally, Daniel Fuller, Kevin G Stanley, Daniel Rainham

**Affiliations:** Saskatchewan Population Health and Evaluation Research Unit, University of Saskatchewan, Saskatoon, SK Canada; Community Health and Epidemiology, University of Saskatchewan, Saskatoon, SK Canada; School of Public Health, University of Saskatchewan, Saskatoon, SK Canada; Computer Science, University of Saskatchewan, Saskatoon, SK Canada; Environmental Science, Dalhousie University, Halifax, NS Canada

## Abstract

**Background:**

Children can be highly active and highly sedentary on the same day! For instance, a child can spend a couple of hours playing sports, and then spend the rest of the day in front of a screen. A focus on examining both physical activity and sedentary behaviour throughout the day and in all seasons in a year is necessary to generate comprehensive evidence to curb childhood obesity. To achieve this, we need to understand where within a city are children active or sedentary in all seasons. This active living study based in Saskatoon, Canada, aims to understand the role played by modifiable urban built environments in mitigating, or exacerbating, seasonal effects on children’s physical activity and sedentary behaviour in a population of children in transition from preadolescence to adolescence.

**Methods/Design:**

Designed as an observational, longitudinal investigation this study will recruit 800 Canadian children 10–14 years of age. Data will be obtained from children representing all socioeconomic categories within all types of neighbourhoods built in a range of urban designs. Built environment characteristics will be measured using previously validated neighbourhood audit and observational tools. Neighbourhood level socioeconomic variables customized to Saskatoon neighbourhoods from 2011 Statistics Canada’s National Household Survey will be used to control for neighbourhood social environment. The validated Smart Cities Healthy Kids questionnaire will be administered to capture children’s behaviour and perception of a range of factors that influence their activity, household (including family socioeconomic factors), parental, peer and neighbourhood influence on independent mobility. The outcome measures, different intensities of physical activity and sedentary behaviour, will be collected using global positioning system equipped accelerometers in all four seasons. Each accelerometry cycle will be matched with weather data obtained from Environment Canada. Extensive weather data will be accessed and classified into one of six distinct air mass categories for each day of accelerometry. Computational and spatial analytical techniques will be utilized to understand the multi-level influence of environmental exposures on physical activity and sedentary behaviour in all seasons.

**Discussion:**

This approach will help us understand the influence of urban environment on children’s activity, thus paving the way to modify urban spaces to increase physical activity and decrease sedentary behaviour in children in all four seasons. Lack of physical activity and rising sedentariness is associated with rising childhood obesity, and childhood obesity in turn is linked to many chronic conditions over the life course. Understanding the interaction of children with urban spaces will reveal new knowledge, and when translated to actions will provide a strong basis for informing future urban planning policy.

## Background

Physical activity’s (PA) benefits in preventing non-communicable diseases and improving psychological wellbeing in children have been well established [1, 2]. Despite this evidence, physical inactivity has reached pandemic levels [3], with a majority of children not accumulating the recommended levels of PA for health benefits [4]. Since behavioural interventions directed at individuals have not consistently produced the anticipated PA increase at the population level, in recent years an interdisciplinary field of study, active living research (ALR), has gained prominence [5, 6]. Rooted in eco-social perspective, active living research encompasses exercise, recreational activities, household and occupational activities, and active transportation [6]. Active living interventions, therefore, aim to influence policy to modify environmental exposures at multiple levels (e.g. built environment, schools), thus facilitating PA change in populations [6].

In active living research, the segregation of sedentary behaviour (SB) from PA is critical; emerging evidence suggests that SB have negative effects on health outcomes, and that these are independent of the benefits of PA [7–9]. Child and adolescent-specific ALR evidence indicates some consistent findings. Safety, perception of recreational environment, and opportunity for active transportation have been positively associated with PA [10–19]. Recent reviews [20, 21] have also highlighted the role of multilevel (home, school and individual level) environmental determinants on PA in children and adolescents. Although a significant focus of ALR in children and adolescents has been on PA alone, emerging evidence suggests a stronger role for home environment, where parental support and family’s higher socioeconomic status being associated with lower SB [20, 22]. In terms of neighbourhood environment, higher perceived safety has been associated with lower SB [23].

One consistent gap in ALR to date is the under-exploration of how built and social environmental factors interact with the variation of weather to influence PA and SB. Studies that have explored seasonal weather variation’s influence on PA (and almost none relating to SB) have focused exclusively on seasonal or weather patterns, instead of the interaction between weather variation (a non-modifiable factor) and built and social environmental exposures (modifiable factors) [24–31]. Thus, ALR needs to move towards longitudinal investigations by incorporating seasonal weather variation along with built and social environmental exposures.

The significance of the influence of weather is especially important in temperate and continental climatic zones such as in Canada and in continental Europe due to its wide seasonal variation [32]. Within Canada, prairie provinces like Saskatchewan are known for particularly extreme variations in seasonal weather [33, 34], and there is evidence to indicate that the relationship between seasonality and PA is stronger in Saskatchewan [30]. This study is part of an ongoing active living research initiative set in the city of Saskatoon, Saskatchewan, Canada – Smart Cities, Healthy Kids (www.smartcitieshealthykids.com). Using generalizable and replicable built environment measurement tools [35–38] and objective measures of PA and SB, we have conducted two cross-sectional investigations in 2010 and 2012 on two different cohorts of children representing all residential neighbourhoods of Saskatoon. These investigations have not only contributed to the growing evidence base such as children accumulating higher moderate to vigorous PA (MVPA) on weekdays and density of destinations being detrimental to children’s higher PA [39], but we have also developed a methodology to minimize measurement bias due to systematic accelerometer wear-time variation [40].

The cross-sectional findings of Smart Cities, Healthy Kids initiative lay the foundation for this longitudinal investigation which aims to study how built and social environmental factors interact with seasonal weather variation to influence PA and SB. This study has three specific objectives.

1. In each of the four seasons in Saskatoon, ***where*** do children accumulate Moderate to Vigorous Physical Activity, Light Physical Activity and Sedentary Behaviour on weekdays and weekend days?
2. Does seasonality determine ***where*** children accumulate MVPA, LPA and SB on weekdays and weekend days? If so, do family, school and built environment characteristics modify seasonality’s effect on location of various level of activity?
3. Does seasonality determine children’s ***overall*** MVPA, LPA and SB accumulation on weekdays and weekend days after taking into account the context location of activity, the family, school and built environment characteristics?

This longitudinal ALR will not only inform local urban planning policy to facilitate the creation of active communities, but will also generate evidence that could be generalized to similar geographic areas across the world.

## Methods/Design

### Study design

Designed as an observational, longitudinal investigation that examines built and social environment’s association with objectively measured GPS-linked PA and SB throughout the year, this study will be conducted in 800 Canadian children who are in transition from preadolescence to adolescence (10–14 years). To capture PA and SB in all seasons in one consecutive 12 month period, after scrutinizing the historical weather data for seasonality [33, 34], these four pairs of months have been selected for data collection: January–February (Winter), April–May (Spring), July–August (Summer) and October–November (Autumn). Data will be collected in the 2014–2015 academic school year. The data collection cycles and main groups of outcome and explanatory variables are outlined in Tables 1 and 2.

**Table 1:** Overview of study variables from current data collection cycles, 2014–2015

Season	Measured variables^a^	Collection modes^b^
*Autumn* October and November 2014	Time-stamped physical activity: moderate to vigorous (MVPA), light physical activity (LPA)	Accelerometry
	Time-stamped sedentary behaviour (SB)	Accelerometry
	Weekday and weekend MVPA, LPA, and SB	Accelerometry
	Sleep duration (in minutes)	Accelerometry
	Time-stamped location and velocity	GPS data loggers
	Self-reported location of activity	Log sheet
	Weather (spatial synoptic classification system)	Environment Canada database
	Body mass index (BMI)	Directly measured height and weight
	Individual and family characteristics (e.g., socio-demographics, activities)	Questionnaire
	School characteristics (general, physical activity facilitators)	Questionnaire
*Winter* January and February 2015		
*Spring* April and May 2015		
*Summer* July and August 2015		

^a,b^Measured variables and collection modes will be the same for each cycle (Autumn, Winter, Spring, Summer)

**Table 2:** Overview of study variables from previous data collection

Context	Measurement tool	Derived variables
Neighbourhood built environment characteristics	Neighbourhood Active Living Potential [35, 36]	Density of destinations, activity friendliness, safety and universal accessibility
	Irvine Minnesota Inventory [37, 38]	Diversity of destinations, attraciveness, safety from crime and traffic and pedestrian acess
Neighbourhood and household social environment characteristics	Statistics Canada Census 2006 and Statistics Canada Household Survey 2011	Median household income, neighbourhood deprivation index, household income,mother’s and father’s education

### Ethics

The project has received ethical approval from the University of Saskatchewan’s Human Behavioural Research Ethics Board (#14–83, approval date: March 27, 2014), which follows the Canada’s Tri-Council Guidelines for Ethics in Research, and governance clearance from the two participating school divisions in Saskatoon, Canada.

Informed consent has been be obtained from all study participants’ parents/guardians. Both parents/guardians have received detailed information outlining study goals and requirements. We have been sensitive to any ethical implications of participants using the GPS data loggers and have taken due care to inform participants and their parents/guardians regarding the purpose and the manner in which the data will be collected, used and secured.

### Urban design

**Fig. 1:**
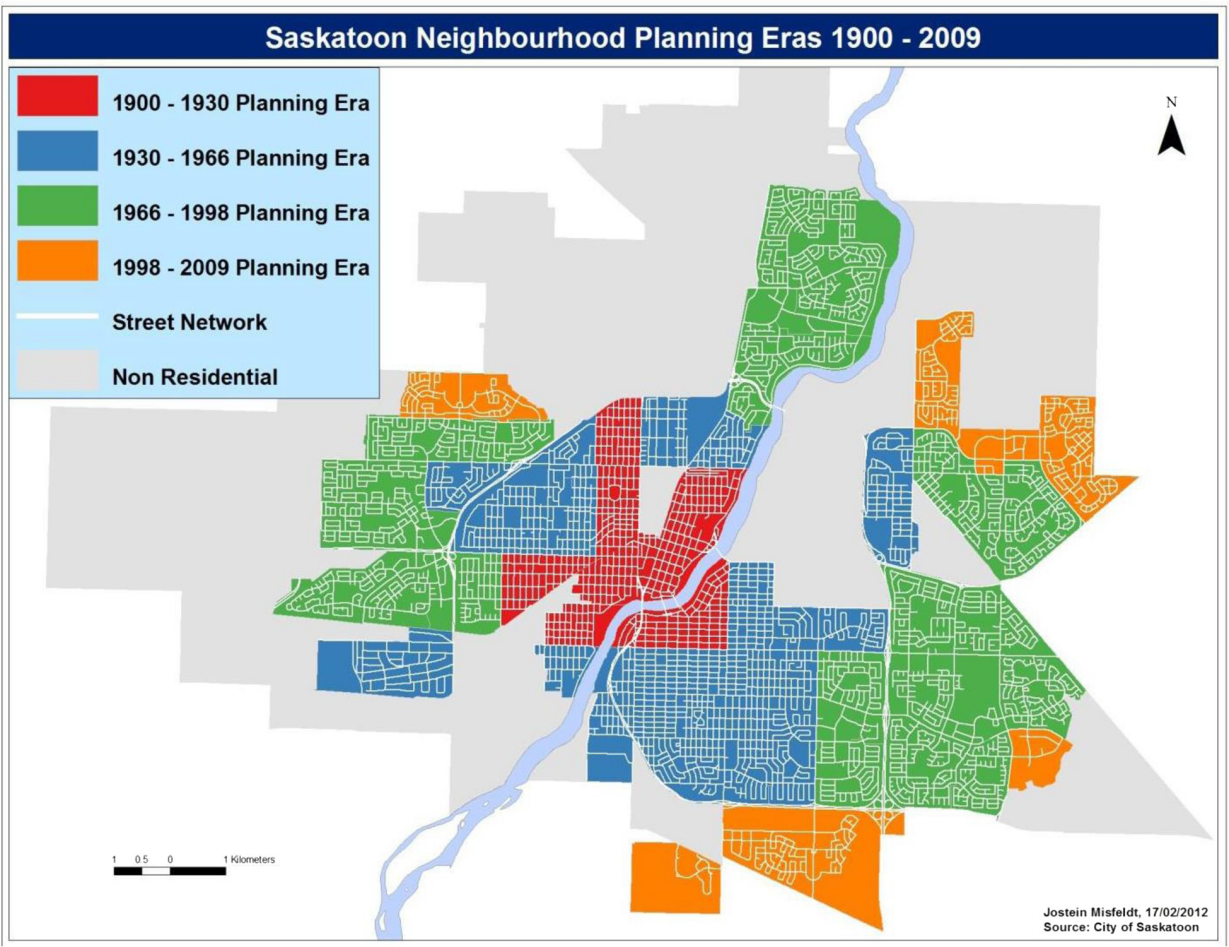
Neighbourhood planning eras, 1900–2009, Saskatoon, Saskatchewan, Canada

Presently the city of Saskatoon’s metropolitan area population of 260,600 is spread across 65 residential neighbourhoods [41]. Saskatoon has the 2^nd^ lowest population density, and 4^th^ highest cumulative metropolitan sprawl rank amongst Canada’s 27 largest cities [42]. Saskatoon is developed in well-defined neighbourhoods where the city plays a major role in the planning and designing of neighbourhoods, the assignment of locations of services and amenities, and the creation of elementary schools. The neighbourhoods designed prior to 1930 follow a traditional grid-patterned street design (Fig. 1), typified by higher density, mixed-use neighbourhoods connected by straight, intersecting streets and back alleys.

The semi-suburban neighbourhoods built between 1931 and 1966 follow a fractured grid-pattern. They are predominantly residential, with lower density and become progressively car-oriented as the distance from the urban centre increases. Finally, the suburban neighbourhoods built after 1967 follow curvilinear street patterns, characterized by low-density, almost exclusively residential and highly car-oriented configurations. Working with the City of Saskatoon’s Neighbourhood Planning Department, our research team has adopted the urban designs predominant in the different eras of urban development in Saskatoon [43].

### Neighbourhood selection, participant recruitment and sample size

The sampling frame for recruiting children consists of all residential neighbourhoods in Saskatoon categorized into the three socioeconomic categories (low, moderate, and high). This categorization was based on Pampalon deprivation index [44] derived from 2010 Generation five census projection data [45] aggregated to each Saskatoon neighbourhood. Our goal is to obtain data from children representing all socioeconomic categories within all types of neighbourhoods. A sample of 800 provides adequate power while maximizing cost efficiency to detect an effect size of 0.4 with an α of 0.05 in four measurement periods. The sample is adequately powered to conduct subgroup analysis by urban design with 80 % power to detect an effect size of 0.4 with an α of 0.05. As we expect an attrition rate of about 25–30 % over 1 year of data collection, we will recruit 1100 children initially. This strategy was used to recruit children in spring of 2012 for cross-sectional component of Smart Cities Healthy Kids study. Recruitment will occur through elementary schools in each selected neighbourhood by identifying intact classes for recruitment (four classrooms at each elementary school, from Grades 5 to 8).

### Built environment

Based on previously validated tools with generalizable evidence, two replicable tools have been used to measure the built environment ─ Neighbourhood Active Living Potential [35, 36] and Irvine-Minnesota Inventory [37, 38] (Table 2). Neighbourhood Active Living Potential is an 18 item tool that has been validated by the Smart Cities Healthy Kids team [46] which consists of dimensions, safety, density of destinations and activity friendliness, and an added dimension universal accessibility (measures disabled individuals’ access to built environment). Pairs of observers independently rated neighbourhoods by travelling a predetermined route created by random selection of block segments and connecting them to complete a walking route. The inter-observer reliability was above 80 % [46]. Similarly, two observers were employed to administer the Irvine Minnesota Inventory (inter-observer reliability of above 70 %), to measure BE in five dimensions, diversity of destinations, pedestrian access, attractiveness, and safety from traffic and safety from crime. In both tools, safety is measured as the observers’ perceived safety of the neighbourhoods.

### GPS-equipped accelerometry and anthropometry

Hip-mounted triaxial accelerometers (Actigraph GT3X+) will be deployed through schools in the four pairs of months selected for data collection. Participants will be visited at their respective schools and instructed on how to wear the accelerometer equipped belt to maintain proper positioning (i.e. posterior to the right iliac crest of the hip) [47] every day for seven consecutive days (including sleeping hours), unless entering water. Participants will be asked to return the accelerometers at the end of the 7 day cycle. The devices will be programmed to measure data at 00:00 on the day following device deployment (i.e., almost a full day after the device was deployed) to minimize the potential for subject reactivity within the first day of wearing the accelerometer. Moreover, during each cycle of accelerometry, to calculate body mass index, we will measure height and weight of each participant.

Accelerometer generated data will be analyzed using ActiLife 6 data analysis software (Version 6.11.4, ActiGraph Corp., Pensacola, FL). Accelerometer data will be collected in 100 Hz epochs. Valid data (at least four valid days, with each day having 10 h of recorded accelerometry data) will be standardized using methods described by Katapally *et al.* [40]. Accelerometry data will be analyzed at 1 s epochs and reduced by defining a series of activity intensities using cut-points specific for the study sample’s age group ─ SB: <100 cpm; light PA (LPA): 100 to <1500 cpm; MVPA: ≥1500 cpm [48–50]. These activity intensities represent the complete range of daily waking activity. Biologically implausible data (>15,000 cpm) [51], including non-wear time (60 min. epochs with <2 min. interruptions of continuous 0 s) will be removed from data inputs on a case-to-case basis [52].

The objectively measured PA and SB data will be matched with their location of accumulation obtained my QStarz Bt-1000XT bluetooth-enabled GPS data loggers [53]. The hip-mounted GPS data loggers will be deployed along with the accelerometers during each seven day accelerometry period. These devices will record location and velocity data every second. To determine compliance with using both the accelerometers and the GPS data loggers, each participant will be given a seven days compliance log to complete daily. Ultimately, by creating a neighbourhood destination index, participants’ PA and SB accumulation will be matched with a range of locations both within and outside their neighbourhoods. (A detailed presentation of accelerometry data collection methods is available in a manual form, on request [54].)

### Neighbourhood socioeconomic environment

Previously derived neighbourhood level socioeconomic variables customized to Saskatoon neighbourhoods from 2011 Statistics Canada’s National Household Survey will be used to control for neighbourhood social environment.

### School environment

Before accelerometers are deployed, at the time of preparing the schools for data collection, we will use a previously employed questionnaire to collect data about school environment (e.g. indoor and outdoor recreational facilities, as well as school policies that determine opportunities for PA and SB).

### Individual and household environment

Previously utilized Smart Cities Healthy Kids questionnaire will be administered to participants prior to the first accelerometer deployment to capture participants’ behaviour and perception of a range of factors that influence their activity. These factors encompass household (including family socioeconomic factors), parental, peer and neighbourhood influence on independent mobility and ultimately, PA and SB.

### Seasonality

Saskatoon experiences a continental climate with four distinct seasons, average temperatures of 3.4 °C in spring, 17.2 °C in summer, 3.2 °C in autumn, and −14 °C in winter, relatively low levels of precipitation, and predominantly northwestern winds of 15 km/h year round [55, 56]. With temperate summers, and cold winters, the city remains under snow cover for an average of 6–7 months annually. To capture this wide variation in weather, we will employ the spatial synoptic classification methodology [57].

Humans respond to the complete range of weather conditions acting in concert simultaneously at given time [57] rather than to any one specific weather element. The spatial synoptic classification methodology [57] recognizes the range and variety of localized weather elements rather than measuring individual variables such as temperature and precipitation. Studies have used synoptic methods to confirm associations between respirable particles and mortality [58], to estimate the impact of climate change on human health [59], to aid in the forecast of air pollution episodes [60], and to explore air pollution effects on human mortality during extreme weather conditions [61].

The spatial synoptic classification system-2 [57] is an advanced version of this approach which is designed to classify complex daily weather conditions into one of six distinct air mass categories, and a transitional category which represents a day when one weather category yields to another. These categories include: dry moderate; dry polar, dry tropical, moist moderate, moist polar, moist tropical, and transitional. The categories are defined according to several weather variables including dry-bulb temperature, dew point, u (East–west) and v (North–south) components of wind, cloud cover, and sea level pressure collected at four different times of the day [57]. We will use the synoptic classification to assign a weather category to each day of accelerometry.

### Analyses

The main outcome variables will be MVPA, LPA and SB. These outcome variables will be matched with time stamped location of participants using a GIS-based toolbar, the Geo Activity Processor [62]. Matched GPS weekday and weekend activity data will be imported into a GIS and locations of MVPA, LPA and SB will be identifıed using a combination of street network, municipal cadastral data, satellite imagery, and an enhanced points-of-interest fıle developed by TeleAtlas [63].

Locations (for example, school, home, commute between school and home, etc.) of weekday and weekend MVPA, LPA and SB will be identified and ranked based on the percentage of activity accumulated at each location. Categories of ranking will be established to determine major locations of weekday and weekend activity accumulation. Rankings will be categorized by seasons for all participants, across and within seasons for participants in different neighbourhood type, and across seasons for participants within the same neighbourhood type. These patterns will be mapped over the city of Saskatoon to visualize the relationship between specific exposures and 10–14 year old children’s activity. In each category of rankings, variation of MVPA, LPA and SB between the same major locations will be tested by a combination of paired and independent t-tests.

After identifying the major locations of weekday and weekend accumulation of each activity intensity (MVPA, LPA and SB), longitudinal multilevel multivariate responses models [64, 65] will be fitted (separate for weekday and weekend activity). The multivariate responses multilevel model will allow us to consider all the three outcomes (MVPA, LPA and SB) in a single model to account for the full range of activity intensities in an integrated manner. The data will be considered as comprising four levels (level 1 = time/seasons, level 2 = individual/family characteristics, level 3 = school characteristics, level 4 = neighbourhood characteristics). The time-varying predictor variables at level one will include synoptic weather categories, and age (in months). At level two individual and household/family characteristics will be included. At level three, school characteristics will be introduced. At level four, urban design, and neighborhood level built and social environment variables will be introduced as described above. Cross-classified multilevel models between the school and neighbourhood levels will be used so as to simultaneously consider school and neighbourhood characteristics [65–67]. Relevant interaction terms for variables within and between levels (cross-level) will be created to test specific hypotheses and assess how the accumulation of MVPA, LPA and SB at major locations across seasons is modified by neighbourhood and school environment. Results based on a similar approach utilizing cross-classified multilevel models have been reported by our team previously [68–70].

## Discussion

This paper describes the methodology of a comprehensive active living study that aims to understand how built and social environmental factors modify the influence of weather variation on objectively measured PA and SB in children in transition from preadolescence to adolescence. An innovative contribution of the study is the usage of GPS devices in delineating the factors that determine the accumulation of PA and SB in different urban spaces in different weather patterns throughout a year. Understanding the interaction of children with urban spaces will reveal new knowledge, and when translated to actions will provide a strong basis for modifying urban design. In essence, GPS integration with accelerometry will allow the identification of specific locations that are frequented by children during times of specific activity levels, thus providing a unique lens to their independent mobility and the association between environmental factors and objectively measured activity in children.

Established active living evidence thus far indicates the role of multilevel (urban design, home, school) environmental determinants on PA in children and adolescents [10–21]. However, the emergence of SB as a factor having negative effects on health outcomes that are independent of the benefits of PA [7–9] has added an additional layer of complexity to Active Living Research. To date, most studies investigating the influence of environment on SB have utilized disparate and narrowly defined measurement methods (i.e., screen time, self-reports) [20, 22, 23]. Realizing the importance of using consistent and generalizable methods, we aim to understand how both PA and SB interact with each other within the spatial and social contexts in which they occur.

The analytical approach in this study will benefit from our team’s interdisciplinary expertise which includes experts in epidemiology, kinesiology, health geography, and spatial and computational epidemiology. Regression models will take into account the hierarchical structure of the data by aggregating the children’s data to their respective residential neighbourhoods. Models will utilize all three intensities of activity (MVPA, LPA and SB) as the outcome variables. Although current PA guidelines for children aged 5–11 years and youth aged 12–17 years recommend accumulation of at least 60 min of MVPA every day [71], on any given day, individuals can accumulate this recommended quantity of MVPA, and still remain sedentary for the most parts of the day [72]. Therefore, it is necessary to study SB exclusively [72–74]; Current SB guidelines recommend limiting prolonged sitting, including screen time, and propose frequent interruption of SB [75, 76]. Yet, MVPA and SB do not constitute the full range of daily activity; light physical activity (e.g. household chores, standing and walking) is an essential part of daily activity as well. The Canadian Society for Exercise Physiology recommends taking a ‘whole day’ approach to healthy, active living [77] by achieving or exceeding recommended MVPA every day, and at the same time minimizing SB and maximizing LPA.

## Conclusion

With this study, our aim is to influence urban planning policy by translating research evidence to relevant stakeholders. Knowledge translation would be a key aspect of the study, where we intend to disseminate findings to urban planners, schools and parents to influence a multi-level change in the approach to active living. Ultimately, the dissemination of results will contribute to the growing body of evidence on active living in children globally. The contributions of this study will range from employment of novel methods to the conceptualizing of PA and SB within the contexts they occur. The objective capture of not only the quantity of PA and SB, but also their location, will pave the way for methodologically robust investigations in future. Finally, in terms of conceptualization, our inclusive approach to measuring the full range of activity and sedentariness (MVPA, LPA and SB) complements the comprehensive ecological perspective guiding the Active Living Research practiced today.

## Abbreviations

ALR: Active living research
GIS: Geographical information systems
GPS: Geographical positioning systems
LPA: Light physical activity
MVPA: Moderate to vigorous physical activity
PA: Physical activity
SB: Sedentary behaviour
